# Mr. Lloyd George's Amending Bill

**Published:** 1913-06-28

**Authors:** 


### THE INSURANCE ACT.

### Mr. Lloyd George's Amending Bill.

On Tuesday last the Chancellor of the Exchequer introduced a Bill to amend the Insurance Act. We take from his speech those passages more nearly affecting institutional and medical interests.

The Chancellor of the Exchequer said that in the Bill there was no proposal which would touch the main structure of the National Insurance Act. The first of the main provisions was intended to redeem a pledge given by the Prime Minister to obtain statutory authority for the additional grants made at the beginning of the year for medical benefits. There would be another clause to redeem a pledge given to the medical profession early this year or late last year regarding voluntary contributors, who stopped insurance if their income exceeded £160. The medical profession were very desirous that such people should be excluded from the arrangement with regard to medical benefits.

They had provisions dealing with workers over 50 years of age. It was now proposed to sweep away all those distinctions and to pay the employed contributors who entered into insurance within one year of the Act all benefits up to 70, whatever their age. Medical benefit had also been practically impossible for persons over 65 years of age, but it was now proposed to give all insured persons full medical benefits to the end of life. There had been a difficulty with regard to uninsured members of friendly societies. Those people had been uninsured because they were over 65 years of age, or were disabled when the Act came into operation. They were members of friendly societies and were receiving medical benefit, but it was generally on a basis of 4s. per head, and doctors now regarded that as inadequate, as they were receiving considerably more under the National Insurance Act. They proposed that in future the 2s. 6d. per head which was given in respect of insured persons should be extended to all members of friendly societies who were in receipt of medical benefit before the Act came into operation. There was another part of the Act which they proposed to amend. The House would recollect that certain persons who were employed were exempt from insurance for one reason or another. He believed the number was about 80,000. He now proposed that medical and sanatorium benefits should be provided for them. Another amendment dealt with arrears during unemployment. Before the Act, even during sickness, in the vast majority of cases a member's subscriptions were deducted from his benefit. When he returned, if he was unemployed he had to pay the whole of his arrears. Under the Act a man was allowed four weeks a year. There were no arrears during the time of sickness, but there was a provision that a man would have to make up not only his own but his employer's contribution. He proposed that henceforth a man should only be responsible for his own contribution and not for that of his employer. He felt he had covered pretty well the whole of the amendments which were to be proposed.

To meet the cost of these amendments they proposed first of all to extend the sinking fund from eighteen and a half to twenty years, and in addition they proposed that there should be a contribution from the State. During this year that contribution would amount to £65,000, but in a full year it would come to £207,000.

